# Assessing the Individual Needs and Goals of Dementia Caregivers: Development of the COPE-ING Measure

**DOI:** 10.1093/geroni/igaf122.1568

**Published:** 2025-12-31

**Authors:** Alyssa Aguirre, Jared Benge, Rovianne Rovianne

**Affiliations:** The University of Texas at Austin Dell Medical School, Austin, Texas, United States; The University of Texas at Austin, Austin, Texas, United States; The University of Texas at Austin, Austin, Texas, United States

## Abstract

The experiences of caregivers supporting persons living with dementia (PLWD) vary widely, yet existing measures often fail to accurately identify and address their unique needs and goals. This study aimed to develop the COPE-ING (Caregiver Outcomes of Psychotherapy Evaluation, Individualized Needs and Goals); an assessment tool designed to capture the diverse priorities of dementia caregivers in clinical settings. In the initial stage, we refined 16 items and sought input from 28 caregivers and 24 professional experts to evaluate item importance and provide qualitative feedback. All items were deemed important and revised accordingly. A final version was administered to 100 caregivers representing eight types of dementia, including 40% non-spousal caregivers, 18% non-white participants, and 25% male caregivers. Analysis revealed significant heterogeneity in caregivers’ top priorities, with no single item being prioritized by fewer than 5% of participants. Top priorities included strategies to improve caregiver mood, finding community activities for PLWD, and increasing dementia knowledge. Importance ratings varied, with dementia knowledge, caregiver mood support, and communication strategies being rated highest. These findings outline the initial development of the COPE-ING, a novel measure designed to capture the individualized needs and goals of dementia caregivers. Our findings challenge the assumption that caregivers with similar clinical profiles have similar needs. The COPE-ING tool offers promise for identifying caregiver needs rapidly in clinical settings, with implications for theory and practice, particularly in tailoring interventions and informing caregiving support strategies. Further research is needed to validate the tool across diverse populations and settings.

